# Emerging and Re-Emerging Viral Infections: An Indian Perspective

**DOI:** 10.7759/cureus.30062

**Published:** 2022-10-08

**Authors:** Nandkishor J Bankar, Ashwini A Tidake, Gulshan R Bandre, Ranjit Ambad, Jagadish G Makade, Dattu V Hawale

**Affiliations:** 1 Microbiology, Jawarhal Nehru Medical College, Datta Meghe Institute of Medical Sciences (Deemed to be University), Wardha, IND; 2 Microbiology, Datta Meghe Medical College, Datta Meghe Institute of Medical Sciences (Deemed to be University), Nagpur, IND; 3 Biochemistry, Datta Meghe Medical College, Datta Meghe Institute of Medical Sciences (Deemed to be University), Nagpur, IND; 4 Community Medicine, Datta Meghe Medical College, Datta Meghe Institute of Medical Sciences (Deemed to be University), Nagpur, IND

**Keywords:** epidemic, viral infections, integrated disease surveillance programme, public health emergency of international concern, pandemic, outbreak, reemerging, emerging

## Abstract

Emerging and re-emerging viral infections pose a constant threat, especially in healthcare settings. Viral infections can be thought of as an ecological system, like a forest or a pond, with different species competing for resources. Pandemics tend to occur when there is a disruption to this ecosystem, such as introducing a strain of virus into humans or animals that they have no immunity against. Around 60% of human infectious diseases and 75% of emerging infections are zoonotic, with two-thirds originating in wildlife. There is an ongoing risk of viral diseases as the human population continues to grow and the rate of urbanization increases. The emergence and re-emergence of viral diseases are influenced by a variety of virologic and environmental factors. These factors can be roughly categorized as affecting humans, the environment and/or ecology, and viruses. The spread of zoonotic diseases among humans can be prevented by reducing the transmission risk associated with wildlife and exotic pets through education, legislation, and behavioral change programs that target individuals at risk for exposure.

## Introduction and background

The human immune system is incredibly complex, and no one understands it completely. Scientists are still learning more about our immune response every day, and new findings continue to change the way we think about viral diseases.

A virus is a small microorganism that must infect other cells to replicate. When a virus enters the human body, the immune system responds by producing antibodies, neutralizing it, and preventing transmission from the infected person [1]. However, some viruses have evolved and developed ways to evade detection by the human immune system, allowing them to spread undetected until they reach epidemic levels [2]. Emerging infections are those that are newly discovered or recently rediscovered after being absent for a long period. Re-emerging infections are those that once were common but have become much less frequent as a result of changes in public health practices or an increase in human population density. The severe acute respiratory syndrome (SARS)-associated coronavirus (SARS-CoV-2) epidemic and the human immunodeficiency virus (HIV) pandemic are two classic examples of newly developing infectious diseases that the general population had not before encountered in the 1980s and 2003, respectively. A recurring infection is referred to as "re-emerging." Influenza and pandemics in 1918, 1957, and 1968 are illustrative cases of re-emerging diseases [3].

Viruses with a positive-sense single-stranded RNA (+ss RNA) genome, which affect millions of people worldwide, are responsible for several human diseases, and unfortunately, there is no treatment available for many of these viruses. Dengue virus (DENV), chikungunya virus (CHIKV), and Zika virus (ZIKV) are human +ssRNA viruses that are currently having a substantial effect on public health [4]. As opposed to the aforementioned viruses, the Middle East respiratory syndrome-coronavirus (MERS-CoV) kills about 35% of patients who are diagnosed with the disease [2]. Other RNA viruses that are currently of interest are the Ebola and Marburg filoviruses [5].

Emerging and re-emerging viral infections pose a constant threat, especially in healthcare settings. As the use of immunosuppressive drugs increases, more patients have weakened immune systems. The result is an increase in the rate of transmission of these viruses. While there’s no surefire way to prevent the spread of these viruses, there are measures that can be taken to reduce the risk [6].

This review aims to discuss and highlight emerging and re-emerging viral infections and the factors responsible for their spread in India.

## Review

Epidemics of viruses that cause human disease are widespread, but the reasons why they occur in some populations and not others remain unclear. It is known that more than 200 different virus species can infect people. Yellow fever virus was the first of these to be identified, and three to four new species are still being discovered each year [7]. Viral infections can be thought of as an ecological system, like a forest or pond, with different species competing for resources. Pandemics tend to occur when there is a disruption to this ecosystem, such as introducing a strain of virus into humans or animals that they have no immunity against. Infection with one virus can provoke an immune response that makes another virus more likely to spread throughout the body. Conversely, infection with one type of virus may make another type more dangerous by weakening the body’s defense against it [7,8]. Understanding how these factors affect the spread of viruses has implications for predicting and preventing future pandemics.

Estimates indicate that around 60% of human infectious diseases and 75% of emerging infections are zoonotic, with two-thirds originating in wildlife [9,10]. The zoonotic origin of emerging infections in humans is due to the close interaction between humans and wildlife, including domesticated animals. A major concern for public health is that the emergence and increasing prevalence of zoonotic diseases will have a negative impact on human health, animal welfare, and the economy. Compared to non-zoonotic viral diseases, zoonotic diseases cause more loss in terms of lives and the economy. For example, the Ebola virus was responsible for approximately 11,300 deaths and USD 2.2 billion in economic loss in 2014, whereas rabies is responsible for approximately 59,000 human deaths and USD 8.6 billion in economic loss each year [10].

An additional threat to human health from wildlife is posed by exotic pets. The exotic pet trade has increased dramatically in recent years, and a large percentage of these pets are wild animals that are captured from the wild or bred in captivity [11]. These animals often carry infectious disease agents, which can be transmitted to humans through bites, scratches, or other forms of physical contact with infected animals. The outbreak of monkeypox in pet prairie dogs and the rapid spread of the West Nile virus in North America have both been significant public health occurrences that have highlighted the need for tighter cooperation between the veterinary profession, wildlife experts, and public health workers [12].

The spread of infectious diseases is also facilitated by the increasing global trade in food products, which has led to international food-borne disease outbreaks (e.g., avian influenza). Many zoonotic agents are also found in food products (e.g., hepatitis E virus), while others are spread by vectors that rely on food sources (e.g., mosquitoes) [13].

Emerging and re-emerging viral diseases in India are a major cause for concern. There is an ongoing risk of viral diseases as the human population continues to grow and the rate of urbanization increases. With more than 1.4 billion people residing in India, there is also a high risk of contracting new viral diseases. We are seeing a rise in viral disease due to climate change, which leads to extreme temperature differences during the year.

The Integrated Disease Surveillance Programme (IDSP) in India has identified and categorized viral outbreaks from 2008 to 2020 This is shown in Table 1.

**Table 1:** Major viral outbreaks that have occurred in India from 2008 to 2020. Source: Integrated Disease Surveillance Programme (IDSP) [14].

Viral disease classification	Causative viral agents	Outbreaks
Public Health Emergency of International Concern (PHEIC)	Avian Influenza	All over India except Andhra Pradesh, Tamil Nadu, Arunachal Pradesh, Nagaland, and Mizoram
	COVID-19 (coronavirus disease)	All over India
	Crimean Congo hemorrhagic fever	Gujarat, Rajasthan, Kerala, and Utter Pradesh
	Nipah	Kerala
	Zika	Madhya Pradesh, Gujarat, Tamil Nadu, and Kerala
Zoonotic Diseases	Kyasanur Forest Disease (KFD)	Maharashtra, Goa, Karnataka, Tamil Nadu, Kerala
Vaccine-preventable diseases (VPD)	Measles	All over India
	Mumps	All over India except Chattisgarh, Tamil Nadu, Andhra Pradesh, and most of the North Eastern States
	Polio	Uttar Pradesh, Madhya Pradesh, Bihar, Assam, Uttarakhand, and Tamilnadu
	Rubella	All over India except Chattisgarh, Tamil Nadu, Andhra Pradesh, Telangana, Mizoram, Tripura, and Nagaland
Vector-borne diseases (VBD)	Chikungunya	All over India except Jammu and Kashmir, Punjab, Himachal Pradesh, Chattisgarh, Meghalaya, Tripura, Mizoram, Manipur, Nagaland, and Sikkim.
	Dengue	All over India
	Japanese encephalitis (JE)	All over India except Jammu and Kashmir and Punjab
Water-borne diseases (WBD)	Hepatitis A	All over India except Uttar Pradesh, Bihar, Jharkhand, Sikkim, Manipur, and Nagaland
	Hepatitis E	All over India except Jharkhand, Sikkim, Andhra Pradesh, Manipur, and Nagaland

Recently, on the 19th week of the IDSP report 2022, an outbreak of norovirus was reported from the Palakkad district of Kerala [14].

Contributing factors to emerging and re-emerging viral diseases

The emergence and reemergence of viral diseases are influenced by a variety of virologic and environmental factors. There is no particular, stable ecological niche that viruses occupy. Rather, viruses have the potential to parasitize different host species because of their inherent ability to genetically modify and the evolvability of fitness levels. Selection operates on complex and phenotypically different populations of viruses that are created through mutation, recombination, genome segment reassortment, and combinations of these molecular events [15].

These factors can be roughly categorized as affecting humans, the environment and/or ecology, and viruses.

Human Factors: Population Growth, Urbanization, and Migration

People in developing countries are rapidly migrating from rural areas to cities in search of employment and opportunity. Population distribution is shifting worldwide due to urbanization, colonization, labor associated with agriculture, and mining [16]. Large communicable disease outbreaks may overwhelm the primary and public health systems as a result of the rapid migration of people into urban areas and the creation of slum areas without adequate shelter, clean water, or sanitation. [17]. Overcrowding in urban areas, especially due to poor housing conditions, facilitates the spread of tuberculosis, other respiratory infections, and vaccine-preventable diseases.

Urbanization can be a positive contributor to public health by providing access to essential health services. However, in crowded urban areas without adequate housing and sanitation, infectious diseases spread rapidly due to a lack of proper hygiene practices. In addition, the lack of safe water supplies and sewage disposal systems can result in contaminated water supplies, which are a major contributing factor to emerging and re-emerging infections. India has the largest dengue burden, with one-third of all new infections worldwide. Urbanization, globalization, and a lack of mosquito control are a few reasons that have significantly contributed to the escalation of this viral infection [18,19]. Rapid population growth can overwhelm immunization efforts, reduce herd immunity, and make society more vulnerable to epidemics. Infectious disease has influenced and is influenced by population dynamics, from the plague and smallpox to more recent influenza and SARS outbreaks [20].

Urban areas are potential hotspots for the rapid spread of infectious diseases like SARS and avian flu because of population density and close interaction among residents [21]. Urban areas provide a conducive environment for the spread of a global pandemic, which might cause a global health disaster. Global health and the epidemiology of infectious diseases are faced with a number of issues as a result of urbanization. New megacities may serve as the breeding grounds for new epidemics, and zoonotic illnesses may spread more quickly and pose hazards on a global scale. Appropriate city planning and surveillance can be effective instruments for enhancing overall health and reducing the burden of communicable illnesses [22].

Predisposing socioecological risk factors include an increase in livestock populations, intense interspecies interactions, and widespread ecological change. A classic example is buffalopox in Western Maharashtra, a zoonotic disease that has been linked to numerous animal epidemics and human cases in India. The dynamics of the disease suggest a comparatively high level of transmissibility between animals and humans, and the drivers of rising incomes, urbanization, and globalization may help it spread further [23,24].

Of all new infectious diseases that have been identified since 1940, more than 60% are zoonotic in nature [9]. Hunting for "exotic meat" and living close to domesticated animals can both increase the danger of an infectious disease spreading from the animal host to the people. Significant deforestation brings humans, bats, and even monkeys closer together, which could lead to the transmission of "new" infections like influenza, COVID-19, and MERS. To control and avoid this impending threat to world health, a better understanding of zoonotic disease surveillance, prevention, and management would be extremely valuable. Hotspots for this transmission have been identified, and they frequently correspond to regions where urbanization is clearly on the rise.

Significant food-borne diseases caused by noroviruses and the hepatitis A virus have been linked to food-handler transmission and sewage-contaminated foods. A single food item may include a complex mixture of viruses and other pathogens, which could lead to genetic recombination or reassortment and further increase the diversity of these diseases [25].

Environmental Factors

There is a global and institutional aspect to the environmental problem and its connection to the emergence and reemergence of diseases [26]. The ecosystem, which is made up of biotic and abiotic components that interact in some places, is the fundamental component of the environment. Humans maintain artificially altered ecosystems to meet their own food demands or to supply food production chains [27]. These agrosystems are fragile because they lack the species diversity required to maintain dynamic equilibrium. Animals are accused of causing environmental, infectious, and metabolic annoyances [28]. However, in a globalized society, the health risks linked to newly emerging and re-emerging infections are substantially greater, and recent epidemics like the H5N1/H7N9 avian flu, Rift Valley fever, and Ebola virus illnesses are examples of this [26].

The ecology of an area can also be a contributing factor. Environmental changes such as deforestation or pollution can increase viral exposure through ecological disruption. The emergence of the Ebola virus in West Africa is attributed to deforestation and the subsequent loss of forested areas that are critical for fruit bat populations. Simultaneously, human population growth has led to increased contact between humans and fruit bats [29-31]. Deforestation has also been linked to the re-emergence of the Nipah virus in Malaysia and Singapore since 1998, and increased interactions with pig farms have been linked to the re-emergence of Rift Valley fever virus in Sudan in 2007 and outbreaks in Saudi Arabia in 2000 and Kenya in 2006 [32-35]. Changes in ecology can also lead to increased pathogen exposure via changes in host behavior. For example, increased contact between bats and livestock due to anthropogenic encroachment on bat caves has been linked to the emergence or re-emergence of the rabies virus [13,36].

Impact of Mass Gatherings and Emerging Viral Infections

One of the largest human mass gatherings on Earth is the holy Kumbh Mela, which takes place every 12 years in Uttar Pradesh, India [37]. Also, there are various gatherings taking place in India like the Pushkaram festival in Andhra Pradesh, Velankanni, the biggest Catholic pilgrimage, etc. During such massive gatherings, the transmission of respiratory and gastrointestinal illnesses continues to be a major issue. Several of these gatherings have seen outbreaks of infectious diseases, most illustrated by the cholera epidemic at the Kumbh Mela celebration in 1817, which led to the Asiatic cholera pandemic (1817-1844) due to the returning pilgrims who were infected [6]. These mass gatherings pose a threat to emerging and re-emerging viral diseases because they may serve as a platform for the exchange of genetic material, may be responsible for the evolution of new viruses and may infect people who are susceptible to that particular virus. Although MERS-CoV infection has not been detected in the country, yes, there is a risk of it through international travel, including the Hajj pilgrimage. Reports suggest that returning pilgrims who are infected spread influenza [38].

The emergence, re-emergence, and transmission of viral illnesses may also be influenced by a variety of socioeconomic factors. Inadequate public health infrastructures, restricted immunization programs, and political upheaval or wars that uproot millions of people and leave overcrowded refugee populations without access to clean water or basic medical treatment are a few of these issues [39].

Viral Factors

Viruses are also changing. At least some emerging viruses are genetically evolving more rapidly than before due to mutations that arise more readily under conditions such as faster replication rates caused by insect-borne transmission [34,40] or immunosuppression from HIV infection; some viruses may even be undergoing evolutionary adaptation through natural selection [41]. Viral evolution may also be driven by viral-host co-evolutionary dynamics: as hosts develop resistance to viruses, some viruses may respond by evolving new ways to overcome host defenses [42]. Viruses may also evolve more rapidly through genetic recombination, which is a major mechanism for the evolution of influenza viruses in particular [43]. RNA viruses particularly have high mutation rates, which help them in rapid evolution and environmental adaptation, which in turn maintain equilibrium with their host [3].

The genetic variation of the viral genome is primarily caused by three processes: point mutation, recombination, and reassortment. Point mutation is a frequent adaptation method utilized in the evolution of viruses. The poor fidelity of reverse transcriptase (RT), which lacks the 3′-5′ proofreading exonuclease, and RNA-dependent DNA polymerase (RDDP) is the cause of the high mutation rate of RNA viruses [44]. Recombination is the second method used by viruses to adapt through mutation. Recombination creates a new "mixed" or "hybrid" genome molecule by allowing two copies of genetic information to interact. Coronaviruses exhibit a high rate of recombination [45,46]. Gene reassortment is the third mechanism of viral adaptability. When segmented viruses, which are viruses with numerous segmented genomes, coinfect the same cell, gene reassortment takes place, ultimately resulting in the progeny virus having a genome set generated from the various parent viruses [3].

Some emerging viruses have been found to be more virulent than their ancestral counterparts, such as the pathogenic H5N1 avian influenza virus, which has already caused a pandemic in poultry. It keeps infecting humans and other mammals across species boundaries, frequently with catastrophic results and fatal outcomes [47]. Others seem less virulent: the 1918 pandemic strain of the influenza virus killed around 50 million people worldwide, with a case fatality rate of >2.5%, whereas recent pandemic strains have been estimated to kill less than 1% [48-50], although this could be in part due to differences in viral load between then and now. Still, other emerging viruses are now being transmitted by novel means. Person-to-person transmission of the Middle East respiratory syndrome coronavirus, for example, was previously thought to be impossible due to the virus's preference for respiratory epithelium. However, cases have recently been reported with probable transmission from human to human via large droplets from coughing or sneezing [51].

When the newly emerging COVID-19 virus is compared with the SARS (severe acute respiratory syndrome) virus and MERS (Middle East respiratory syndrome) coronavirus, case fatality was quite lower in COVID-19 as compared to SARS (9.5%) and MERS (34.4%). This was attributed to the basic reproductive number (R0) of COVID-19 (2.0-2.5), which was higher than SARS (1.7-1.9) and MERS (<1) [52].

**Table 2:** Important emerging and re-emerging viruses in India Source: Integrated Disease Surveillance Project (IDSP), World Health Organization (WHO), Centre for Disease Control (CDC)

Sr No	Family	Disease	Virus	Recent emergence/ re-emergence	The state where the emergence or re-emergence was detected
1	Paramyxoviridae	Encephalitis	Nipah virus	2001	West Bengal
2	Rhabdoviridae	Encephalitis	Chandipura virus	2003	Andhra Pradesh
3	Togaviridae	Fever and arthralgia	Chikungunia virus	2003	Andhra Pradesh
4	Picornaviridae	Neurological disease and hand, foot, and mouth disease in children	Human enterovirus 71	2003	Kerala
5	Hantaviridae	Hemorrhagic fever with renal syndrome (HFRS) and hantavirus cardiopulmonary syndrome (HCPS)	Hantavirus	2005	Tamil Nadu
6	Orthomyxoviridae	Acute respiratory disease	H1N1 influenza virus (Swine flu)	2009	Andhra Pradesh
7	Bunyaviridae	Hemorrhagic fever	Crimean-Congo hemorrhagic fever virus	2011	Gujarat
8	Flaviviridae	Microcephaly, congenital nervous system malformations, and Guillain-Barré syndrome	Zika virus	2017	Gujarat
9	Coronaviridae	Acute respiratory disease	SARS COV-2	2020	Kerala
10	Flaviviridae	Hemorrhagic fever	Kyasanur forest disease virus	2021	Karnataka
11	Orthomyxoviridae	Acute respiratory disease	H5N1 Influenza virus (avian influenza)	2021	Haryana
12	Poxvirus	Flu-like symptoms and rash	Monkeypox virus	2022	Kerala

The importance of epidemiology

In the late 19th century, a new scientific discipline, epidemiology, emerged that focused on the emergence of disease and traced its causes and effects in populations at risk. This field has grown over the past century into several branches including ecologic and social epidemiology, molecular epidemiology, and public health surveillance [53]. It is currently used by public health workers to prevent disease outbreaks and reduce their impact on human populations. Epidemiologists monitor infectious diseases in populations by collecting surveillance data from cases diagnosed with a specific disease or condition over time [54]. This information can be used to track an epidemic's source and transmission routes, estimate its future spread, predict when it might end, guide public health interventions (such as vaccination programs), identify high-risk groups (by demographics or exposure), estimate numbers of people who might be affected in future outbreaks (for example, predicting how many people might be infected if an outbreak occurs), guide research into the causes and treatments of diseases, improve understanding of how diseases work (their "mechanism" or natural history), develop new diagnostic tests for diseases, establish quarantine measures for suspected carriers before they spread the disease further, etc.

Although epidemiology provides useful information about disease emergence in humans, some emerging infectious diseases defy control using epidemiological methods. Rapid and sometimes unexpected changes in the patterns of transmission of infectious diseases have occurred. An example is HIV/AIDS. Although HIV emerged more than half a century ago, epidemiologists could not predict the pathogen's global spread until several decades after its initial emergence because it took years for this disease to be recognized as a global health problem, even though it had been spreading across continents for decades [55]. It was too late to slow down this epidemic using existing epidemiological methods. Furthermore, epidemiologists are limited in how far back in time they can analyze diseases because historical records are incomplete or inadequate for monitoring diseases that were not considered important at the time.

## Conclusions

The spread of zoonotic diseases among humans can be prevented by reducing the transmission risk associated with wildlife and exotic pets through education, legislation, and behavioral change programs that target individuals at risk for exposure. Public health officials should also work closely with veterinarians to improve surveillance for emerging diseases among domestic animals that may be sentinel species for early detection of new infectious agents in wildlife populations that could pose a threat. The primary focus should be on improving outbreak response capacity and strengthening diagnostic laboratory networks for the early detection of emerging and re-emerging viral diseases.
